# Parental Migration and Left-Behind Children’s Depressive Symptoms: Estimation Based on a Nationally-Representative Panel Dataset

**DOI:** 10.3390/ijerph15061069

**Published:** 2018-05-24

**Authors:** Mi Zhou, Xiaotong Sun, Li Huang, Guangsheng Zhang, Kaleigh Kenny, Hao Xue, Emma Auden, Scott Rozelle

**Affiliations:** 1 College of Economics and Management, Shenyang Agricultural University, Shenyang 110866, Liaoning, China; zhoumisyau@163.com (M.Z.); sunxiaotong1994sk@163.com (X.S.); 2 School of Business, Liaoning University, Shenyang 110031, Liaoning, China; 3 Rural Education Action Program, Freeman Spogli Institute for International Studies, Stanford University, Stanford, CA 94305, USA; krkenny@stanford.edu (K.K.); eauden@126.com (E.A.); rozelle@stanford.edu (S.R.); 4 School of Economics and Management, Northwest University, Xi’an 710069, Shanxi, China; xuehjjx@gmail.com; 5 Center for Experimental Economics in Education, Shaanxi Normal University, Xi’an 710127, Shanxi, China

**Keywords:** depressive symptoms, left-behind children, human capital, PSM-DID

## Abstract

China’s rapid urbanization in the past several decades have been accompanied by rural labor migration. An important question that has emerged is whether rural labor migration has a positive or negative impact on the depressive symptoms of children left behind in the countryside by their migrating parents. This paper uses a nationally representative panel dataset to investigate whether parental migration impacts the prevalence of depressive symptoms among left-behind children in China. Using DID and PSM-DID methods, our results show that parental migration significantly increases the depression scores of 10 and 11-year-old children by 2 points using the CES-D depression scale. Furthermore, we also find that the negative effect of decreased parental care is stronger than the positive effect of increased income in terms of determining the depressive symptoms status of children in rural China.

## 1. Introduction

In China, despite rapid economic growth and a huge concentration on children’s cognitive skills, many rural children have not only developed better cognitive skills in China’s competitive schooling system, but have also developed serious mental health problems at the same time, such as depression. In one province sample of China, 25% of all children display symptoms of depression [1]. Other research has shown that, in 2012, 20.3% of Chinese children aged 10–15 years displayed symptoms of depression, and this share was even higher among Chinese children from rural areas (23%) [2]. According to the 6th national census, there are 39.20 million children in rural areas, which means nearly 9.02 million rural children display symptoms of depression.

The fact that such a large number of rural children are at risk for depression has negative implications not only for life-long individual economic and social outcomes, but also China’s future human capital development. For example, poor mental health and depressive symptoms are associated with low health-related quality of life [3]. In particular, children with mental health problems or depressive symptoms can lead to dropout among junior high students [4]. The depressive symptoms of children could have negative societal impacts later in life, such as crime, decreased earnings, and poor health and education outcomes [5,6]. Left-behind children (LBCs), in particular, are more likely to develop depressive symptoms than children of the same age from non-migrant families in China [7].

Evidence seems to suggest that parental absence contributes to children’s depressive symptoms, and leads to more severe depression outcomes among LBCs as compared to children living with their parents. For example, research has found that negative impacts on children’s depressive symptoms when their primary caregiver is absent [8,9,10]. This situation can arise for the following reason: parental migration leads to disruptions in children’s relationships with their parents [11], reduces communication with their parents [12].

Interestingly, however, there are also studies that suggest that there is little difference in depressive symptoms outcomes between the LBCs and other children living in rural areas. Some studies even find that LBCs display better depressive symptoms outcomes than their peers, especially in terms of schooling outcomes [13,14,15]. This situation can arise when remittance income improves the financial situations of migrant households and more resources are invested in LBCs, which could prevent or alleviate depressive symptoms.

Given this mixed evidence, the goal of this paper is to determine whether parental migration positively or negatively impacts the depressive symptoms outcomes of children in rural China. In pursing this research goal, we examine the overall effects of parental migration on the depressive symptoms of children and explore the mechanisms through which these impacts occur. Specifically, these mechanisms include increased household income and decreased levels of care received by LBCs. To do so, this paper uses a propensity score matching difference-in-difference (PSM-DID) model to analyze the causal effect of parental migration on the depressive symptoms of LBCs.

The rest of this article is organized as follows: Section 2 describes our data and methodology, including the sampling methods, data collection, and methodology. Section 3 presents our results on the effect of parental migration on the depressive symptoms outcomes of LBCs. Section 4 examines the mechanisms through which parental migration affects LBC depressive symptoms (namely, increased income and decreased care). Section 5 discusses our findings and concludes.

## 2. Data

### 2.1. Data Source and Sampling

The data used for this study come from the China Family Panel Studies (CFPS) for the years 2010 and 2014. The CFPS is a nationally representative, longitudinal social survey that was launched in 2010 and is conducted biennially by the Institute of Social Science Survey (ISSS) at Peking University, China. The survey design is based on the Panel Survey of Income Dynamics (PSID), the National Longitudinal Surveys of Youth (NLSY), and the Health and Retirement Study (HRS) in the United States. It focuses on a range of topics related to education outcomes, economics activities, migration, health, and family dynamics. The survey collects data at three levels: the individual-, family-, and community-levels.

The CFPS surveyed respondents in sampling units in 25 provinces (all provinces except Xinjiang, Tibet, Qinghai, Inner Mongolia, Ningxia, and Hainan), a sampling frame which represents 95% of the Chinese population. To generate nationally and provincially representative sample, the CFPS adopted a “Probability-Proportional-to-Size” (PPS) sampling strategy with multi-stage stratification and carried out a three-stage sampling process. The first stage was the Primary Sampling Unit, in which county-level units were randomly selected. In the second stage, village-level units (villages in rural areas and neighborhoods/communities in urban areas) were selected. In the third stage, households from the village-level units were selected according to the study’s systematic sampling protocol. All members of each household were interviewed, except those who were not at home at the time of the survey.

**Figure 1:**
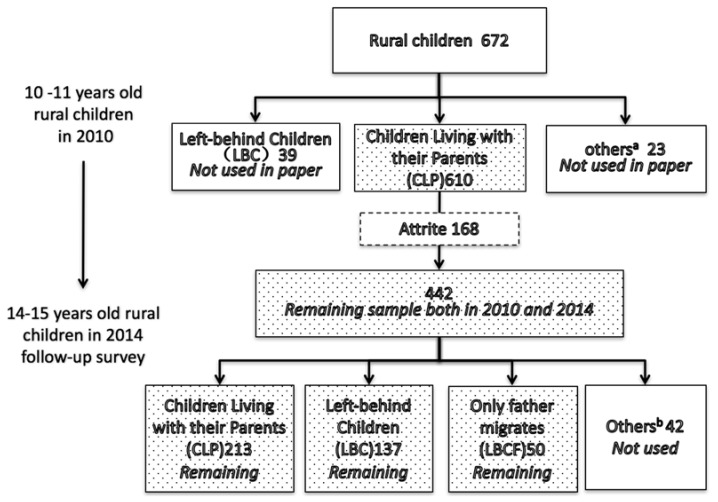
The construction of panel data. Source: CFPS (2010, 2014); ^a^ Others include: only father migrates or only mother migrates; ^b^ Others include: migrant children either with Hukou or without Hukou; ^c^ The shaded boxes show the division of the three main subgroups in our sample.

Following an initial baseline survey wave in 2010, ISSS conducted two follow-up surveys in 2012 and 2014. For the purposes of the survey, we examine the case of children who were 10 and 11-year-old at the time of the 2010 survey and 14 and 15-year-old, respectively, at the time of the 2014 survey. We limit our sample to children between the ages of 10 and 15 because children between these ages responded to the child questionnaire and were of the age to respond to the survey independently. In this study, we use the CFPS data from 2010 and 2014 to create a panel dataset that includes 442 children for both years, after excluding observations with missing information (Figure 1). Because the 2010 and 2014 CFPS data both include the same depression scale (CES-D), this panel allows us to examine the depressive symptoms outcomes of children over these two periods. For the sake of our analysis, we exclude an additional 42 children whose parental migration status did not fit into one of our sample classifications. In the end, our sample is comprised of 400 children (we have added a balance test to identify whether there are systematic differences between the 168 children who dropped out and the 442 who remained in the sample. Just as Appendix Table A3 shows, we generally did not find systematic differences between these two groups).

We also created three “types” of rural children: left-behind children (LBC), who have rural hukous and had resided in rural areas while both of their parents work and live outside of the household for at least 5 months out of the previous year; father-only migration children (LBCF), who have rural hukous and had resided in rural areas with their mother while only their father works and lives outside of the household for at least 5 months out of the previous year (only a small portion of our sample were “mother-only migration children” (2.84% of the sample), and for this reason we do not evaluate this group of children); and children living with both parents (CLP) in their rural communities.

Figure 1 shows that, among the 400 children in our sample who were living with their parents in 2010, at the time of the 2014 CFPS survey 213 children living with both parents, and are classified as *children living with parents* (CLPs); 137 were living with neither parent, and are classified as *left-behind children* (LBCs); and 50 were living with only their mother, and are classified as *father-only migrates children* (LBCFs) (for our analysis, we exclude children from households where only the mother migrated (*n* = 15) or with any other type of household migration status (*n* = 27, total) due to their small sample size).

### 2.2. Measures

The depression scale used for the 2010 and 2014 CFPS data is the Center for Epidemiologic Studies Depression Scale (CES-D). This scale was developed by Radloff [16] and has been used widely in the international literature [17,18]. The CES-D includes 20 items that are designed to evaluate four aspects of depression (somatic symptoms, depressed affect, positive affect, and interpersonal problems). If more than four responses are missing from a single observation, then it was dismissed. Due to changes in the scale between the time of the 2010 and 2014 CFPS surveys, some items in the CES-D scale did not match up between the two years. Specifically, six of the items in the scale matched between the two years, so we evaluate changes in depression status on these six items and dismiss the other 14 (see Appendix Table A1). Due to the small group of value 1, we replace value 1 to 2, and then use 5 minus the score of each question to get the adjusted score, which range from 0 to 3. The higher score means the more severe of depressive symptoms.

### 2.3. Descriptive Analysis

As can be seen in Table 1, 52.49% of children in our sample are male (row 3, column 2) and 11.76% are non-Han cultural minorities (row 7, column 2). In terms of the age distribution, 50.23% of respondents were 11 years old at the time of the 2010 CFPS survey, and 15 years old at the time of the 2014 CFPS survey (row 10, column 2). In 2010, the fraction of students that boarded at school was 12.67% (row 15, column 2), and this proportion increased to 53.17% by 2014 (row 15, column 5). We also found that the self-report health status of children worsened over our study period, as the share of children that reported “excellent” health fell from 75.34% in 2010 (row 17, column 2) down to 28.51% in 2014 (row 17, column 5).

In terms of individual-level characteristics, we find that there are significant differences between LBCs and CLPs in terms of school boarding status, household income, and self-reported health status in 2014 (Table 2). Specifically, LBCs are more likely to board at school than CLPs, significant at the 5% level (Row 4, Column 4); LBCs have average household per capita incomes 301.73 yuan higher than CLPs, significant at the 1% level (Row 5, Column 4); and LBCs have measures of self-reported health 0.19 higher than CLPs, significant at the 5% level (Row 6, Column 4).

**Figure 2:**
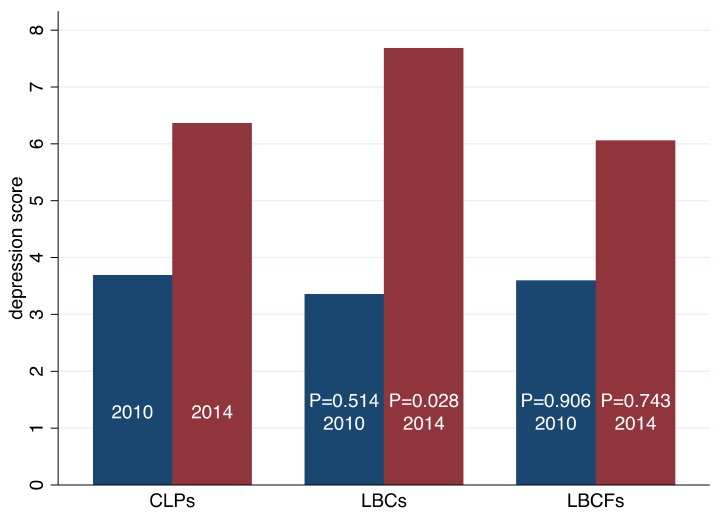
Distribution of depression score between 2010 and 2014 among three main subgroups. Source: CFPS (2010, 2014). The *p*-value of difference test of depression score between CLPs and LBCs’ in 2010 is 0.514. The *p*-value of difference test of depression score between CLPs and LBCFs’ in 2010 is 0.906. The *p*-value of difference test of depression score between CLPs and LBCs’ in 2014 is 0.028. The *p*-value of difference test of depression score between CLPs and LBCs’ in 2014 is 0.743.

When examining the change in depression scores of our sample of 442 children, we find that the average depression score increases from 3.54 in 2010 (Table 1, Row 1, Column 3) to 6.98 in 2014 (Row 1, Column 6) significantly at 1% level. To examine whether changes in parental migration status may have contributed to this increase in average depression scores, we compare the depression scores of CLPs to those of LBCs/LBCFs in 2010 and 2014 (Figure 2).

We find that there is both insignificant difference between CLPs and LBCs in 2010, and between CLPs and LBCFs in 2010. However, there is a significant difference (at the 5% level) between CLPs and LBCs in 2014, but there is no significant difference between CLPs and LBCFs in the same year.

## 3. Methodology

### 3.1. Difference-in-Difference Approach

We employ a Difference-in-Difference (hereafter, DID) approach to compare the outcomes (i.e., depression scores) of students in the treatment group (that is, children who were CLPs in 2010 and LBCs/LBCFs in 2014) before and after the parent(s) out-migrated to children in the comparison group (that is, children who remain CLPs). This comparison produces what we call standard DID estimator. The model we estimated is restricted and unadjusted model:

(1) $$\Delta score_{i}=\alpha +\beta \cdot Mig_{i}+\lambda \cdot C_{c}+\varepsilon_{i}$$

where i denotes a child in the sample, and $\Delta score_{i}$ is the change in depression score of child i between baseline survey and endline survey (that is, the endline CES-D score minus the baseline CES-D score of the same child *i*). $Mig_{it}$ is the treatment variable, and β is the parameter of interest? In our analysis, we have two different treatments, as discussed above, namely: LBCs from households where both parents migrated and LBCFs from households where only the father. The county effect is captured by the coefficient λ.

In addition to the standard DID estimator [19], we implemented three other DID estimators: an “unrestricted” version that includes baseline outcomes as a right-hand variable, an “adjusted” version that includes other covariates in addition to the treatment variable, and an unrestricted/adjusted model that combines the features of both the “unrestricted” and “adjusted” model. The unrestricted and adjusted DID estimators relax the implicit restrictions in the standard DID estimator that the coefficient associated with baseline outcomes and covariates gathered from baseline survey equals one. The combination of unrestricted and adjusted DID estimators relaxes both assumptions. The models to be estimated are as follows:

The unrestricted and unadjusted model is:

(2) $$\Delta score_{it}=\alpha +\beta \cdot Mig_{it}+\delta \cdot score_{it-1}+\lambda \cdot C_{c}+\varepsilon_{it}$$

The restricted and adjusted model is:

(3) $$\Delta score_{it}=\alpha +\beta \cdot Mig_{it}+\gamma \cdot X_{it}+\lambda \cdot C_{c}+\varepsilon_{it}$$

and, the unrestricted and adjusted model is:

(4) $$\Delta score_{it}=\alpha +\beta \cdot Mig_{it}+\delta \cdot score_{it-1}+\gamma \cdot X_{it}+\lambda \cdot X_{c}+\varepsilon_{it}$$

where the term $X_{it}$ is a vector of covariates that are included to capture the characteristics of children, such as their gender, age, ethnic minority status, school boarding status, annual household income, and self-reported health. $score_{it-1}$ represents the baseline depression score of child i.

### 3.2. Propensity Score Matching Approach

In addition to the set of DID estimators, we also used a matching approach to check and see whether our results are robust to our choice of estimators. Rosenbaum and Rubin [20] proposed Propensity Score Matching (PSM) to reduce the bias in the estimation of treatment effects with observational data sets. PSM allows the analyst to match children in the treatment group (LBCs) with similar children from the control group (all children except LBCs). The difference between their depression scores is the treatment effect, and the average of all children’s treatment effect is the effect of parents’ migration on children’s depressive symptoms.

**Figure A1:**
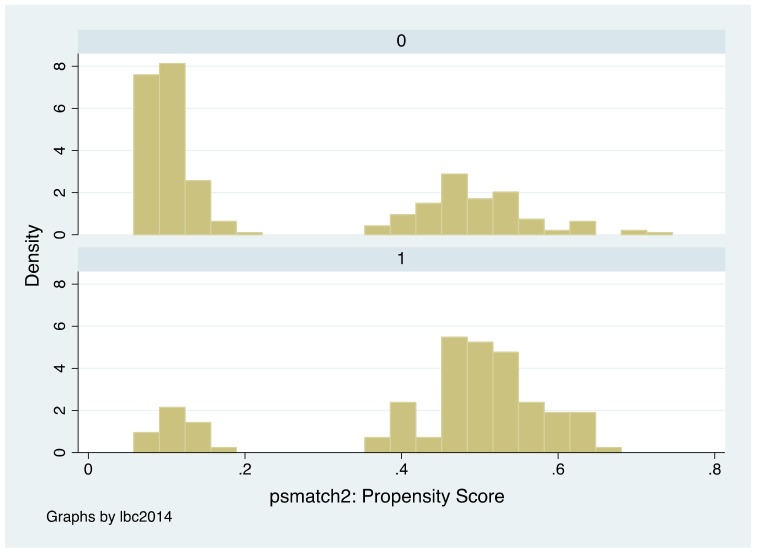
Overlap in the support of the variable between LBC and other children in 2014. Source: CFPS (2014).

To implement the matching estimator successfully, we follow a series of well-established steps [21]. First, we check the overlap in support of the covariates between treatment group and control group. Intuitively, wide common support means that there is a large overlap in the propensity scores. In our study, the common support is wide in our sample (Appendix Figure A1). This means that we can estimate the average treatment effect for the treated sample. In the second step, we choose the method of matching. In this study, we used the nearest neighbor matching method with replacement. The standard errors are bootstrapped using 1000 replications. The last step is to assess the matching quality. Since we do not condition on all covariates but on the propensity score alone in PSM, it should be checked whether the matching procedure can balance the distribution of the relevant covariates in both the treatment and control group. To do so, we use balance tests described in Dehejia and Wahba [22,23]. The balancing tests were satisfied for all covariates.

In order to guard against the potential source of bias [24], we also implemented the Bias-Corrected Matching (BCM) estimator. To minimize geographic mismatch, we enforce exact matching by county. Each treatment observation is matched to three control observations with replacement, which is few enough to enable exact matching by county for nearly all observations but enough to significantly reduce the asymptotic efficiency loss [25]. Matching is based on a set of 7 covariates, including gender, age, ethnic minority status, school boarding status in 2014, the log of family income per person in 2014, self-reported health in 2014, and depression score in 2010 (see Table 1). The weighting matrix uses the Mahalanobis metric, which is the inverse of the sample covariance matrix of the matching variables.

Finally, since all matching methods only match observations based upon observable covariates, they do not account for all unobservable covariates. To control for part of the unobservable factors that are time-invariant, we extended the cross-sectional matching estimator to a longitudinal setting and implemented a Difference-in-Difference Matching estimator (DDM). When implementing DDM, we use both PSM and BCM methods.

## 4. Estimation Results

### 4.1. The Effect of Parental Migration on the Depressive Symptoms Outcomes of LBCs

Our analysis using the panel data set shows that parental migration increases the prevalence of depressive symptoms among LBCs (Table 3). Specifically, when we examine the unrestricted and unadjusted model in Column 1, we find that the depression scores of LBCs increase by 1.955 points relative to those of other children (significant at the 10% level—Row 1, Column 1), but there is no significant impact on the depression scores of LBCFs (Row 2, Column 1). We find similar impacts the unrestricted and unadjusted model in Column 2 (1.872—significant at the 5% level) and the restricted and unadjusted model in Column 3 (2.861—significant at the 5% level), as well as in the restricted and adjusted model in Column 4 (1.973—significant at the 10% level). Again, we find that the coefficient on LBCF variables are insignificant in all models.

However, parental migration may have differential impacts on a child’s depressive symptoms depending on whether or not the migrating parent is of the same gender as the child. For this reason, we added gender and “only father migrates” (LBCF) as control variables in our DID regression equation. However, we found that a child’s gender was not significantly correlated with whether or not the child develops depressive symptoms. In addition, we also examined whether adding an interaction term between gender and LBCF (where only the father migrates) would have an impact on our results, and ultimately, found that the interaction term is insignificant (Row 4, Columns 1–4, in Table 3).

### 4.2. Matching and DID-Matching Results

The results of the cross-sectional matching analysis, regardless of the method of matching, reveal that parental migration has significant positive effect on the depressive symptoms outcomes of students, meaning that their depressive symptoms worsened after their parents migrated.

Specifically, when we estimated the effect of parental migration on the depressive symptoms of LBCs, we can found a coefficient 1.147 (Table 4, Row 1, Column 1) using propensity score method and a coefficient of 2.707 (Row 7, Column 1) using bias corrected matching. We find that our results generally remain the same when we used the DID-matching estimator. Specifically, we find a coefficient of 2.162 (Row 1, Column 2) using propensity score matching and a coefficient of 2.731 (Row 7, Column 2) using bias corrected matching method.

### 4.3. Mechanism Analysis

While we found significant positive impacts of parental migration on the depression scores of LBCs, meaning that the depression symptoms of children worsened after their parents migrated. We believe that these results arise from the competing influences of two effects: a parenting effect and an income effect. The parenting effect refers to the decreased care children receive following the migration or one or both parents, which we anticipate is negative and therefore would have a positive effect on the depression scores of children. The income effect refers to the increased household income that results from remittance income, and we expect this to have a positive impact on children’s depressive symptoms and therefore a negative effect on their depression scores. We use PSM method to examine the parenting and income effects separately.

#### 4.3.1. Parenting Effect

In examining the parenting effect, we look at the cases where only the mother or only the father migrates. We anticipate that there will be differences between these two groups because mothers are typically children’s primary caregivers. Therefore, we expect their absence would result in a larger reduction in care than if the father migrated. And our results show that this appears to be the case. Specifically, Table 5 shows that if a child’s mother migrates, their depression score increases by 1.668 points on average (significant at the 10% level—Row 2).

Meanwhile we find no significant impact on children’s depression scores if their father migrates (Row 3). We also find that when a child’s father had already migrated in 2010 and the child’s mother out-migrated by 2014, we find that depression scores increased by an average of 2.105 points (significant at the 10% level—row 3). These findings suggest that there is a negative care effect on children, but it is not significant if only the child’s father migrates.

#### 4.3.2. Income Effect

In addition to examining the parenting effect on the depressive symptoms of LBCs, we also estimate the income effect using PSM. To do so, we divide children into those from higher income households (meaning, those with household incomes above the mean) and lower income households (with household incomes below the mean) using 2010 data. Then we matched children (that is, LBCs to CLPs) within the higher income and the lower income groups. The results show that there is no significant effect of parental migration on depressive symptoms outcomes when children are matched in this manner (Table 5, Row 6).

Considering that it is likely *increases* in household income that would lead to improvements in children’s depressive symptoms, we also tested whether there were any effects of household income increases on the depression scores of LBCs. To do so, we matched LBCs and CLPs based on whether or not their household income increased at least 1.5 times between 2010 and 2014. Again, we find no significant result on the depressive symptoms outcomes of LBCs (Table 5, Row 7).

Lastly, we compared the depressive symptoms outcomes of CLPs to those of LBCFs. We did this with the understanding that LBCFs are likely to see larger increases in income and smaller decreases in care, and therefore may potentially fare better than CLPs if increased income impacts depressive symptoms outcomes. However, we find no significant impact from this analysis (Table 5, Row 8).

## 5. Conclusions

This paper uses nationally representative panel data to explore the relationship between parental migration and the depressive symptoms outcomes of LBCs. In addition, we use PSM analysis to examine whether these outcomes arise due to the effects of either increased income or decreased care. Our results show that there seem to be significant positive effects of parental migration on the depressive symptoms of LBCs, meaning that children experience worsened depressive symptoms following the migration of their parents. Our results also suggest that this worsening of depressive symptoms outcomes likely occur due to decreased care received by LBCs, and this impact cannot be counteracted by the effects of increased household income. By providing evidence suggesting that parental migration negatively impacts the depressive symptoms outcomes and leads to depressive symptoms among LBCs, this paper suggests that the Chinese government should develop methods to improve the depressive symptoms condition of LBCs.

## Figures and Tables

**Table 1:** Sample distribution.

		2010			2014		
		*n*	%	Depression Score	*n*	%	Depression Score
		[1]	[2]	[3]	[4]	[5]	[6]
[1]	All	442	100	3.54	442	100	6.98
[2]	Gender						
[3]	Male	232	52.49	3.47	232	52.49	7.21
[4]	Female	210	47.51	3.61	210	47.51	6.78
[5]	Minority						
[6]	Han Chinese	390	88.24	3.54	390	88.24	6.71
[7]	non-Han Chinese	52	11.76	3.62	52	11.76	9.06
[8]	Age						
[9]	10	220	49.77	3.43			
[10]	11	222	50.23	3.66			
[11]	14				220	49.77	7.00
[12]	15				222	50.23	6.79
[13]	Did you board at your school?						
[14]	0 = no	386	87.33	3.47	207	46.83	7.16
[15]	1 = yes	56	12.67	4.05	235	53.17	6.83
[16]	How would you rate your health status?						
[17]	1 = excellent	333	75.34	3.20	126	28.51	5.71
[18]	2 = very good	93	21.04	4.41	155	35.07	6.35
[19]	3 = good	10	2.26	3.20	131	29.64	8.47
[20]	4 = fair	5	1.13	10.00	24	5.43	9.25
[21]	5 = poor	1	0.23	9.00	6	1.36	8.50

Source: CFPS (2010, 2014).

**Table 2:** Descriptive statistics of control variables used in the multivariate analysis.

		Total	CLP	LBC		LBCF	
		Mean	Mean	Mean	H0:(3) = (2)	Mean	H0:(5) = (2)
		(s.e.)	(s.e.)	(s.e.)	Difference	(s.e.)	Difference
		[1]	[2]	[3]	[4] ^a^	[5]	[6] ^b^
[1]	Male in 2014, 1 = yes 0 = no	0.47	0.47	0.51	0.04	0.38	−0.09
		(0.50)	(0.50)	(0.50)	(0.05)	(0.49)	(0.08)
[2]	Age in 2014	10.50	14.49	14.49	0.00	14.52	0.03
		(0.50)	(0.51)	(0.52)	(0.00)	(0.50)	(0.01)
[3]	Minority in 2014, 1 = yes 0 = no	0.12	0.99	1.00	0.01	1.00	0.01
		(0.32)	(0.10)	(0.00)	(0.29)	(0.00)	(0.08)
[4]	Board in 2014, 1 = yes 0 = no	0.13	0.38	0.85	0.47 ***	0.38	0.00
		(0.33)	(0.49)	(0.35)	(0.05)	(0.49)	(0.08)
[5]	Family net income per person in 2014	4396.92	7911.53	8213.26	301.73 ***	9767.28	1856.00
		(4647.06)	(9111.72)	(5991.50)	(884.71)	(7707.61)	(1292.00)
[6]	Self-report health in 2014	1.30	2.04	2.23	0.19 **	2.08	0.04
		(0.59)	(0.95)	(0.89)	(0.03)	(0.90)	(0.14)

Source: CFPS (2014). Note: Standard errors in parentheses, *** *p* < 0.01, ** *p* < 0.05; ^a^ The difference between the column (2) and column (3) is calculated by regressions of each of row variables on the dummy variable that represent whether the children is left behind in 2014; ^b^ The difference between the column (2) and column (5) is calculated by regressions of each of row variables on the dummy variable that represent whether only children’s father migrates in 2014.

**Table 3:** Difference in difference regression results analyzing the effects of migration activities of parents on children’s depression scores, China.

Dependent Variable: The Difference of Depression Score (Score_14_-Score_10_)		Restricted & Unadjusted	Unrestricted & Unadjusted	Restricted & Unadjusted	Restricted & Adjusted
		[1]	[2]	[3]	[4]
[1]	LBCs in 2014, 1 = yes 0 = no	1.955 *	1.827 **	2.861 **	1.973 *
		(1.049)	(0.883)	(1.283)	(1.030)
[2]	Male in 2014, 1 = yes 0 = no	0.490	0.512	0.033	−0.009
		(1.022)	(0.888)	(1.003)	(0.860)
[3]	LBCFs in 2014 1 = yes 0 = no	−2.543	−1.868	−3.064	−2.450
		(2.439)	(1.977)	(2.451)	(1.995)
[4]	(LBCFs in 2014) * (Male in 2014)	−0.133	1.945	−0.028	2.053
		(2.375)	(2.040)	(2.510)	(2.006)
[5]	Age in 2014			−1.733 *	−1.290
				(0.966)	(0.851)
[6]	Minority in 2014, 1 = yes 0 = no			−7.182 ***	−10.736 ***
				(1.612)	(2.686)
[7]	Board in 2014, 1 = yes 0 = no			−2.001	−0.842
				(1.464)	(1.233)
[8]	The log of family income per person in 2014 (Yuan)			−0.673 *	−0.266
				(0.396)	(0.376)
[9]	Health self-report in 2014			0.370	0.514
				(0.600)	(0.499)
[10]	Standardized depression report in 2010		−3.417 ***		−3.494 ***
			(0.530)		(0.533)
[11]	Constant	2.510 **	−0.620	44.205 ***	32.414 **
		(1.022)	(1.003)	(13.906)	(12.765)
[12]	County dummy	yes	yes	yes	yes
[13]	Observations	442	442	415	415
[14]	R-squared	0.627	0.722	0.669	0.762

Source: CFPS (2010, 2014). Standard errors in parentheses, *** *p* < 0.01, ** *p* < 0.05, * *p* < 0.1.

**Table 4:** Evaluating the effects of migration activities of parents on children’s depression scores using matching and difference-in-difference matching, China.

Treatment Variable: Left-Behind Children, 1 = Yes 0 = No		Matching	Difference-in-Difference Matching
		[1]	[2]
[1]	Propensity score matching		
[2]	Coefficient	1.147 *	2.162 **
[3]	Std. error	(0.600)	(1.046)
[4]	County dummy ^a^	Yes	Yes
[5]	Control variables ^b^	Yes	Yes
[6]	Bias corrected matching		
[7]	Coefficient	2.707 ***	2.731 ***
[8]	Std. error	(0.901)	(0.898)
[9]	County dummy	Yes	Yes
[10]	Control variables	Yes	Yes

Source: CFPS (2010, 2014). Standard errors in parentheses, *** *p* < 0.01, ** *p* < 0.05, * *p* < 0.1; ^a^ Independent variables include: male in 2014, age in 2014, minority in 2014, board in 2014, The log of family income per person in 2014 (Yuan), Health self-report in 2014 and Standardized depression report in 2010; ^b^ we also calculate the standard error by bootstrap, and the results are almost as same as our reported.

**Table 5:** Parenting effect and income effect in PSM.

Standardized Depression Scores		Coeff.	Std. Error	Obs.	R-Square
		[1]	[2]	[3]	[4]
[1]	Parenting effect				
[2]	Only mother migrate matching all children in 2014 ^a^	1.668 **	(0.653)	415	0.016
[3]	Only father migrate matching all children in 2014	0.751	(0.638)	413	0.003
[4]	LBCF2014 matching LBC2014 ^b^	2.105 *	(1.104)	175	0.021
[5]	Income effect				
[6]	Family income in 2010	−0.669	(0.658)	415	0.002
[7]	Family income increased	−0.182	(0.654)	413	0.000
[8]	CLP2014 matching LBCF2014 ^c^	−0.381	(1.016)	246	0.001
[9]	Both parenting and income effect				
[10]	CLP2014 matching LBC2014 ^d^	1.724 **	(0.721)	327	0.017

Source: CFPS (2010, 2014). Standard errors in parentheses, ** *p* < 0.05, * *p* < 0.1; ^a^ Mother is often her kid’s first care-giver in China, especially in rural areas. In this paper the parenting effect is measured by matching only mother migrates children with other children on the same characters. To compare father’s parenting effect with mother’s parenting effect, we also matched only father migrates children with other children on the same characters (row 3), but the difference between them is insignificant; ^b^ LBCFs and LBCs both have income effect, but the former one have parenting effect comparing to the latter one. So, we match LBCs and LBCFs to measure the parenting effect; ^c^ CLPs and LBCFs both have parenting effect, but the former one do not have income effect comparing to the latter one. So, we match CLPs and LBCFs to measure the income effect; ^d^ LBCs do not have parenting effect but have income effect, however, CLPs do not have income effect but have parenting effect. When we match CLPs with LBCs, we can measure the synthetic effect of parenting effect and income effect.

## Appendix A

**Table A1:** CES-D (6-item) in 2010 and 2014.

Item	During the Past Month:	Scores ^b^				
		A ^a^	B ^a^	C ^a^	D ^a^	E ^a^
1	How often did you feel so depressed that nothing could cheer you up?	1	2	3	4	5
2	How often did you feel nervous?	1	2	3	4	5
3	How often did you feel restless or fidgety?	1	2	3	4	5
4	How often did you feel hopeless?	1	2	3	4	5
5	How often did you feel that everything was an effort?	1	2	3	4	5
6	How often did you feel that life was meaningless?	1	2	3	4	5

Source: CFPS (2010, 2014). ^a^ A means “Almost daily”; B means “Often”; C means “Half of the time”; D means “Sometimes”; E means “Never”. ^b^ The score is the sum of the 6 questions. Possible range is 0–30.

**Table A2:** The Center for Epidemiologic Studies Depression (CES-D) test instrument in 2012.

Item	During the Past Week:	Score ^a^			
		A	B	C	D
1	I was bothered by things that usually don’t bother me.	0	1	2	3
2	I did not feel like eating; my appetite was poor.	0	1	2	3
3	I felt that I could not shake off the blues even with help from my family or friends.	0	1	2	3
4	I felt I was just as good as other people.	3	2	1	0
5	I had trouble keeping my mind on what I was doing.	0	1	2	3
6	I felt depressed.	0	1	2	3
7	I felt that everything I did was an effort.	0	1	2	3
8	I felt hopeful about the future.	3	2	1	0
9	I thought my life had been a failure.	0	1	2	3
10	I felt fearful.	0	1	2	3
11	My sleep was restless.	0	1	2	3
12	I was happy.	3	2	1	0
13	I talked less than usual.	0	1	2	3
14	I felt lonely.	0	1	2	3
15	People were unfriendly.	0	1	2	3
16	I enjoyed life.	3	2	1	0
17	I had crying spells.	0	1	2	3
18	I felt sad.	0	1	2	3
19	I felt that people disliked me.	0	1	2	3
20	I could not get going.	0	1	2	3

Source: Radloff, L.S. (1977). The CES-D scale: A self-report depression scale for research in the general population. Applied Psychological Measurement, 1: 385–401. Note: ^a^ A means “Rarely or none of the time (less than 1 day)”; B means “Some or a *little* of the time (1–2 days)”; C means “Occasionally or a moderate amount of time (3–4 days)”; D means “Most or all of the time (5–7 days)” ^b^ The score is the sum of the 20 questions. Possible range is 0–60. If more than four questions are missing answers, then the CES-D questionnaire is not scored. A score of 16 points or more is considered depressed.

**Table A3:** Balance Test.

Dependent Variable: Attrited (1 = yes, 0 = no)	Coefficient	Standard Error
Standardized depression report in 2010	0.111	(0.219)
Age in 2010	−0.086	(0.332)
minority in 2010, 1 = yes 0 = no	2.686 **	(1.315)
board in 2010, 1 = yes 0 = no	0.514	(0.869)
The log of family income per person in 2010 (Yuan)	0.198	(0.194)
Health self-report in 2010	−0.038	(0.367)
County dummy	Yes	
Observations	577	

Source: CFPS (2010). Standard errors in parentheses, ** *p* < 0.05.
